# Supplementary material for: Evolutionary Kuramoto dynamics unravels origins of chimera states in neural populations
Source: PLoS Comput Biol. 2026 Apr 30;22(4):e1014214. doi: 10.1371/journal.pcbi.1014214 (PMC13152215; doi:10.1371/journal.pcbi.1014214)
Supplement: S1 Appendix — Appendix with two-player game order graphs (section 1), derivation of well-mixed communicative fraction with symmetry breaking (section 2), analysis of selection strength δ influence on fcomm (section 3), and analysis of player strategy vs. phase influence on chimera-like index χ (section 4). (PDF) [file pcbi.1014214.s001.pdf]

Supplementary Information for “Evolutionary Kuramoto dynamics unravels origins of chimera states in neural populations”

Thomas Zdyrski, Scott Pauls, Feng Fu

Contents

1 **Two-player game order graphs** 1

2 **Well-mixed communicative fraction with symmetry breaking** 1

3 **Selection strength** 6

4 **Phase *vs.* Strategy** 8

1 Two-player game order graphs

Figure A shows the order graphs for the 16 symmetric, ordinal two-player games. Each sub-panel shows the payoff order for both the column and row player. The letters correspond to the following payoff matrix

$$\begin{array}{c|c|c|} & C & N \\ C & A & B \\ \hline N & C & D \end{array} \tag{1}$$

The horizontal axis represents the order of row player payoffs, and the red lines represent row player options. The vertical axis represents the order of column player payoffs, and the blue lines represent column player options. The arrows show the direction of increasing payoff for the relevant player, and solid black dots represent Nash equilibria. This figure is adapted from the topological taxonomy order graphs [1].

2 Well-mixed communicative fraction with symmetry breaking

Here, we follow the steady-state communicative fraction derivation in the previous EK study [2] and extend it to include a payoff asymmetry α. To keep the derivation tractable, we only consider the well-mixed case. We will assume a low mutation rate so that any mutation fixates prior to the next mutation. We then consider a population with one communicative species $E = (C, \phi_i)$ and one non-communicative species $F = (N, \phi_j)$. Our first goal is to calculate the (communicative) ρ_E and (non-communicative) ρ_F fixation probabilities of a single E or F invader, respectively. First, we use our α-dependent payoff matrix, to calculate the α-dependent average

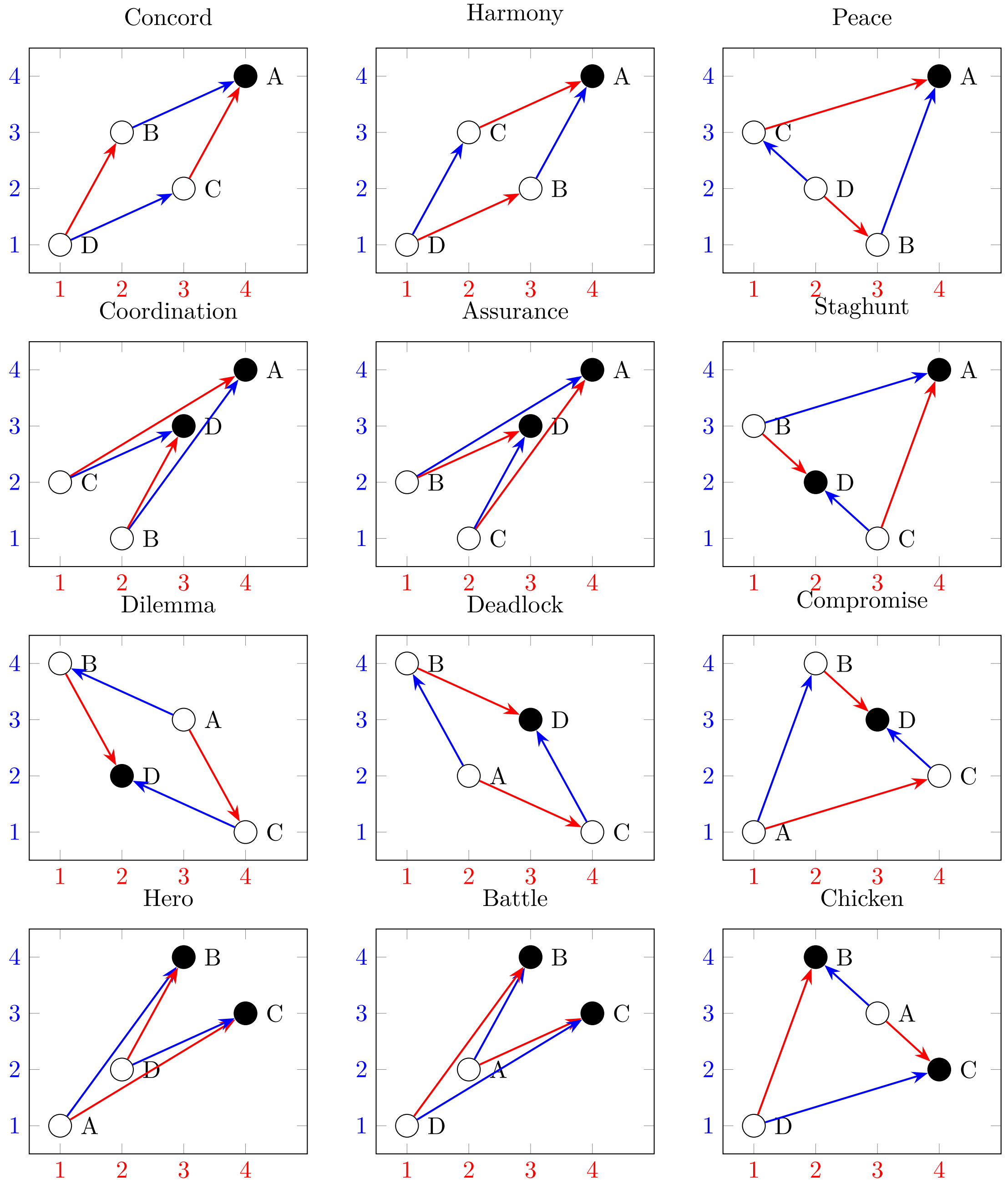

Fig A|Order graphs for the 16 symmetric, ordinal two-player games. Each sub-panel shows the payoff order for both the column and row player. The letters correspond to the payoff matrix in Eq. (1). The horizontal axis represents the order of row player payoffs, and the red lines represent row player options. The vertical axis represents the order of column player payoffs, and the blue lines represent column player options. The arrows show the direction of increasing payoff for the relevant player, and solid black dots represent Nash equilibria. This figure is adapted from the topological taxonomy order graphs [1].

payoff of each strategy across the entire population,

$$\pi_E(k) = \left(\frac{k-1}{n-1}\right)\big(B_0 - c\big) + \left(\frac{n-k}{n-1}\right)\big(\beta(\Delta\phi)2\alpha - c\big) \tag{2}$$

$$= \frac{1}{n-1}\Big(k\big(B_0 - 2\alpha\beta(\Delta\phi)\big) + 2\alpha n\beta(\Delta\phi) - B_0 - (n-1)c\Big) \tag{3}$$

and

$$\pi_F(k) = \left(\frac{k}{n-1}\right)\big(\beta(\Delta\phi)2(1-\alpha)\big) + \left(\frac{n-k-1}{n-1}\right)(0) \tag{4}$$

$$= \left(\frac{k}{n-1}\right)2(1-\alpha)\beta(\Delta\phi) \tag{5}$$

Here, $k \in [0, n]$ is the number of E-players $\Delta\phi$ is the phase difference between the E and F strategies, and we defined $\beta(\Delta\phi) = \beta_0 f(\Delta\phi)$. Note that the first term of π_E corresponds to the E-E payoff, which has $\Delta\phi = 0$, hence $B_0 f(\Delta\phi) = B_0$. As mentioned in the main text, each node has an exponential total fitness $f_E = \exp(\delta(n-1)\pi_E)$ or $f_F = \exp(\delta(n-1)\pi_F)$, depending on its strategy, with selection strength δ.

In this paragraph, we summarize the relevant steps; see the prior work [2] for more detailed derivations. We now model the Moran process as a Markov chain where each state $i \in \{0, 1, \ldots, n\}$ is the number of (communicative) E strategies. Interestingly, we note that since the Moran process changes, at most, one strategy per time step, this Markov chain has the same fixation probabilities as a (state-dependent) "gambler's ruin" problem with ties. We denote x_i as the probability that state i will fixate to state n with all players using strategy E. Then, the E and F fixation probabilities defined above are given by $\rho_E = x_1$ and $\rho_F = 1 - x_{n-1}$. Now, the Markov chain is described by the transition probability $p_{i,j}$ from state i to state j as

$$\begin{aligned}
p_{0,0} &= 1, \\
p_{n,n} &= 1, \\
p_{i,i-1} &= \frac{i}{n}\frac{(n-i)f_F(i)}{if_E(i) + (n-i)f_F(i)} \\
p_{i,i+1} &= \frac{n-i}{n}\frac{if_E(i)}{if_E(i) + (n-i)f_F(i)} \\
p_{i,i} &= 1 - p_{i,i+1} - p_{i,i-1}
\end{aligned}$$

Then, we obtain a recurrence relation for x_i by conditioning on the outcome of the first step:

$$x_i = x_{i-1}p_{i,i-1} + x_i p_{i,i} + x_{i+1}p_{i,i+1}$$

with boundary values

$$\begin{aligned}
x_0 &= 0 \\
x_1 &= 1
\end{aligned}$$

Using the $p_{i,i} = 1 - p_{i,i+1} - p_{i,i-1}$ relation from above to replace the $p_{i,i}$ term gives

$$\big(x_i - x_{i-1}\big)\gamma_i = \big(x_{i+1} - x_i\big)$$

Then, defining $y_i \coloneqq x_i - x_{i-1}$, we find $y_1 = x_1$ and $y_{i+1} = \gamma_i y_i$, yielding $y_i = \prod_{j=1}^{i-1} \gamma_j x_1$ for $i \geq 2$. Finally, we form a telescoping sum to yield

$$1 - x_1 = x_n - x_1 = \sum_{i=1}^{n-1} y_{i+1} = \sum_{i=1}^{n-1} \prod_{j=1}^{i} \gamma_j x_1$$

Solving this for x_1 gives

$$\rho_E = x_1 = \frac{1}{1 + \sum_{i=1}^{n-1} \prod_{j=1}^{i} \gamma_j} \tag{6}$$

Likewise, $x_i = \sum_{j=1}^{i} y_i = \sum_{j=0}^{i-1} y_{i+1}$, so

$$x_i = x_1 + \sum_{j=1}^{i-1} \prod_{k=1}^{j} \gamma_k x_1 = \frac{1 + \sum_{j=1}^{i-1} \prod_{k=1}^{j} \gamma_k}{1 + \sum_{j=1}^{n-1} \prod_{k=1}^{j} \gamma_k}$$

Therefore, we find

$$\rho_F = 1 - x_{n-1} = 1 - \frac{1 + \sum_{j=1}^{n-2} \prod_{k=1}^{j} \gamma_k}{1 + \sum_{j=1}^{n-1} \prod_{k=1}^{j} \gamma_k} = \frac{\prod_{k=1}^{n-1} \gamma_k}{1 + \sum_{j=1}^{n-1} \prod_{k=1}^{j} \gamma_k}$$

This implies

$$\frac{\rho_F}{\rho_E} = \prod_{k=1}^{n-1} \gamma_k \tag{7}$$

Therefore, we've found ρ_E and ρ_F for a population with two fixed strategies.

Now, we need to derive the form of the γ_k, which will include the new asymmetry factor α:

$$\begin{aligned}
\gamma_k &= \frac{f_F(k)}{f_E(k)} \\
&= \exp[\delta(n-1)(\pi_F(k) - \pi_E(k))] \\
&= \exp\left[\delta\Big(k2(1-\alpha)\beta(\Delta\phi) - k\big(B_0 - 2\alpha\beta(\Delta\phi)\big) - 2\alpha n\beta(\Delta\phi) + B_0 + (n-1)c\Big)\right] \\
&= \exp\left[\delta\Big(\big(2\beta(\Delta\phi) - B_0\big)k + B_0 - 2\alpha n\beta(\Delta\phi) + (n-1)c\Big)\right]
\end{aligned}$$

We also calculate

$$\begin{aligned}
\prod_{k=1}^{j} \gamma_k &= \prod_{k=1}^{j} \exp\left[\delta\Big(\Big(2\beta(\Delta\phi_{qr}) - B_0\Big)k + B_0 - 2\alpha n\beta(\Delta\phi_{qr}) + (n-1)c\Big)\right] \\
&= \exp\left[\delta \sum_{k=1}^{j} \Big(\Big(2\beta(\Delta\phi_{qr}) - B_0\Big)k + B_0 - 2\alpha n\beta(\Delta\phi_{qr}) + (n-1)c\Big)\right] \\
&= \exp\left[\delta\left(\Big(2\beta(\Delta\phi_{qr}) - B_0\Big)\frac{j(j+1)}{2} + j\Big(B_0 - 2\alpha n\beta(\Delta\phi_{qr}) + (n-1)c\Big)\right)\right] \\
&= \exp\left[\delta\left(\left(\beta(\Delta\phi_{qr}) - \frac{B_0}{2}\right)j^2 + j\left(\frac{B_0}{2} + \beta(\Delta\phi_{qr})(1-2\alpha n) + (n-1)c\right)\right)\right]
\end{aligned} \tag{8}$$

Additionally, we note that substituting Eq. (8) into Eq. (7) with $j = n-1$ shows that ρ_F/ρ_E depends of $\Delta\phi_{qr}$. This is in contrast to the $\alpha = 0.5$ case [2] where ρ_F/ρ_E simplifies to the $\Delta\phi_{qr}$-independent form $\rho_F/\rho_E = \exp\Big(\delta\big[(n-1)c - B_0(n-2)/2\big]\Big)$.

Next, we consider the long-time dynamics incorporating more than two strategies. By again using the low-mutation-rate assumption, we can assume that any mutation either fixates or dies out before the next mutation. Therefore, the system evolves from one homogeneous state to another using the probabilities we just derived. We can model this behavior with a new Markov chain using the $m = 20$ phases and two communicative strategies (C and N) to give a $2m$-dimensional state space: $\big\{(C,\phi_1),\dots(C,\phi_m),(N,\phi_1),\dots(N,\phi_m)\big\}$. Then, the transition probability $\rho_{NC,\Delta\phi_{qr}}$ from state (N,ϕ_q) to state (C,ϕ_r) is just ρ_E above. Similarly, the probability $\rho_{CN,\Delta\phi_{rq}}$ that a (C,ϕ_r) state is successfully invaded by an (N,ϕ_q) state is simply ρ_F above. By the rotation symmetry of ϕ, each ϕ_i has the same probability of fixation. Therefore, consider an arbitrary ϕ_q and denote its communicative fixation probability as s_1 and its non-communicative fixation probability s_2. We can calculate the ratio of these probabilities by incorporating the fixation probabilities against all other ϕ_r phases:

$$\frac{s_2}{s_1} = \frac{\sum_{r=1}^m \rho_{CN,\Delta\phi_{rq}}}{\sum_{r=1}^m \rho_{NC,\Delta\phi_{qr}}} \tag{9}$$

$$= \frac{\sum_{r=1}^m \rho_{NC,\Delta\phi_{qr}} \exp\Big\{\delta(n-1)\Big[(n-1)c + n\beta(\Delta\phi_{qr})(1-2\alpha) - \frac{n-2}{2}B_0\Big]\Big\}}{\sum_{r=1}^m \rho_{NC,\Delta\phi_{qr}}} \tag{10}$$

$$= \left[\frac{\sum_{r=1}^m \dfrac{\exp\Big\{\delta(n-1)\Big[(n-1)c+n\beta(\Delta\phi_{qr})(1-2\alpha)-\frac{n-2}{2}B_0\Big]\Big\}}{1+\sum_{j=1}^{n-1}\exp\Big\{\delta\Big[\Big(\beta(\Delta\phi_{qr})-B_0/2\Big)j^2+j\Big(B_0/2+\beta(\Delta\phi_{qr})(1-2\alpha n)+(n-1)c\Big)\Big]\Big\}}}{\sum_{r=1}^m \dfrac{1}{1+\sum_{j=1}^{n-1}\exp\Big\{\delta\Big[\Big(\beta(\Delta\phi_{qr})-B_0/2\Big)j^2+j\Big(B_0/2+\beta(\Delta\phi_{qr})(1-2\alpha n)+(n-1)c\Big)\Big]\Big\}}}\right] \tag{11}$$

The first equality used Eq. (7) and the second equality used Eq. (6). Unlike in the $\alpha = 0.5$ symmetric case [2], the ratio ρ_F/ρ_E depends on $\Delta\phi_{qr}$, so we cannot factor the exponential component out of the sum and cancel the $\rho_{CN,\Delta\phi_{qr}}$ terms. However, we can asymptotically expand s_2/s_1 for small $B_0/c \coloneqq \epsilon \ll 1$; additionally, since all of our simulations use $\beta \propto B$, we also assume $\beta/c \sim \epsilon \ll 1$. Finally, to simplify the calculation, we define the asymptotic expansion of $\rho_{NC,\Delta\phi_{qr}} \coloneqq \rho^{(0)}_{NC,\Delta\phi_{qr}} + \rho^{(1)}_{NC,\Delta\phi_{qr}} + \mathcal{O}\Big(\epsilon^2\Big)$ where $\rho^{(i)}_{NC,\Delta\phi_{qr}} \sim \epsilon^i$ depends only on terms of total order i in B_0/c and β/c.

Then, to first order in ϵ we have

$$\frac{s_2}{s_1} = \frac{\sum_{r=1}^{m} \rho_{NC,\Delta\phi_{qr}} \exp\left\{\delta(n-1)\left[(n-1)c + n\beta(\Delta\phi_{qr})(1-2\alpha) - \frac{n-2}{2}B_0\right]\right\}}{\sum_{r=1}^{m} \rho_{NC,\Delta\phi_{qr}}}$$

$$= e^{\delta(n-1)^2 c}\left\{ \frac{\sum_{r=1}^{m}\left(\rho^{(0)}_{NC,\Delta\phi_{qr}} + \rho^{(1)}_{NC,\Delta\phi_{qr}}\right)\left\{1 + \delta(n-1)\left[n\beta(\Delta\phi_{qr})(1-2\alpha) - \frac{n-2}{2}B_0\right]\right\}}{\sum_{r=1}^{m}\left(\rho^{(0)}_{NC,\Delta\phi_{qr}} + \rho^{(1)}_{NC,\Delta\phi_{qr}}\right)}\right\} + \mathcal{O}\left(\epsilon^2\right)$$

$$= e^{\delta(n-1)^2 c}\left\{ \frac{\sum_{r=1}^{m}\left(\rho^{(0)}_{NC,\Delta\phi_{qr}} + \rho^{(1)}_{NC,\Delta\phi_{qr}}\right)}{\sum_{r=1}^{m}\left(\rho^{(0)}_{NC,\Delta\phi_{qr}} + \rho^{(1)}_{NC,\Delta\phi_{qr}}\right)} + \frac{\sum_{r=1}^{m} \rho^{(0)}_{NC,\Delta\phi_{qr}}\delta(n-1)\left[n\beta(\Delta\phi_{qr})(1-2\alpha) - \frac{n-2}{2}B_0\right]}{\sum_{r=1}^{m} \rho^{(0)}_{NC,\Delta\phi_{qr}}}\right\} + \mathcal{O}\left(\epsilon^2\right)$$

$$= e^{\delta(n-1)^2 c}\left\{1 - \delta(n-1)\frac{n-2}{2}B_0 + \frac{\delta n(n-1)}{m}(1-2\alpha)\sum_{r=1}^{m}\beta(\Delta\phi_{qr})\right\} + \mathcal{O}\left(\epsilon^2\right)$$

$$= \exp\left\{\delta(n-1)\left[(n-1)c - \frac{n-2}{2}B_0 + \frac{n(1-2\alpha)}{m}\sum_{r=1}^{m}\beta(\Delta\phi_{qr})\right]\right\} + \mathcal{O}\left(\epsilon^2\right)$$

$$= \exp\left\{\delta(n-1)\left[(n-1)c - \frac{n-2}{2}B_0 + \frac{n(1-2\alpha)}{2}\beta_0\right]\right\} + \mathcal{O}\left(\epsilon^2\right)$$

Where, in the last line, we used the definition of $\beta(\Delta\phi_{qr}) = \beta_0\left[1 + \cos(\Delta\phi_{qr})\right]/2$ to write $\sum_{r=1}^{m}\beta(\Delta\phi_{qr}) = \beta_0\sum_{r=1}^{m}\left\{1 + \cos\left[2\pi(q-r)/m\right]\right\}/2 = m\beta_0/2$ since the cosine sum gives zero.

Finally, since each $\Delta\phi_i$ is equally likely, the probability of fixing to *any* communicative state is ms_1; likewise the total non-communicative fixation probability is ms_2. Then, since the process must eventually absorb to either C or N, we have $ms_1 + ms_2 = 1$, giving the probability of communicative fixation as

$$ms_1 = \frac{1}{1 + s_2/s_1} \tag{12}$$

with s_2/s_1 given by Eq. (11), and the asymptotic, small-ϵ approximation given by

$$ms_1 = \frac{1}{1 + s_2/s_1} \approx \frac{1}{1 + \exp\left\{\delta(n-1)\left[(n-1)c - \frac{n-2}{2}B_0 + \frac{n(1-2\alpha)}{2}\beta_0\right]\right\}} \tag{13}$$

3 Selection strength

The main simulations all used a selection strength of $\delta = 0.2$; this was chosen to be sufficiently small that analytic approximations could be applied (*cf.*, Section 2).

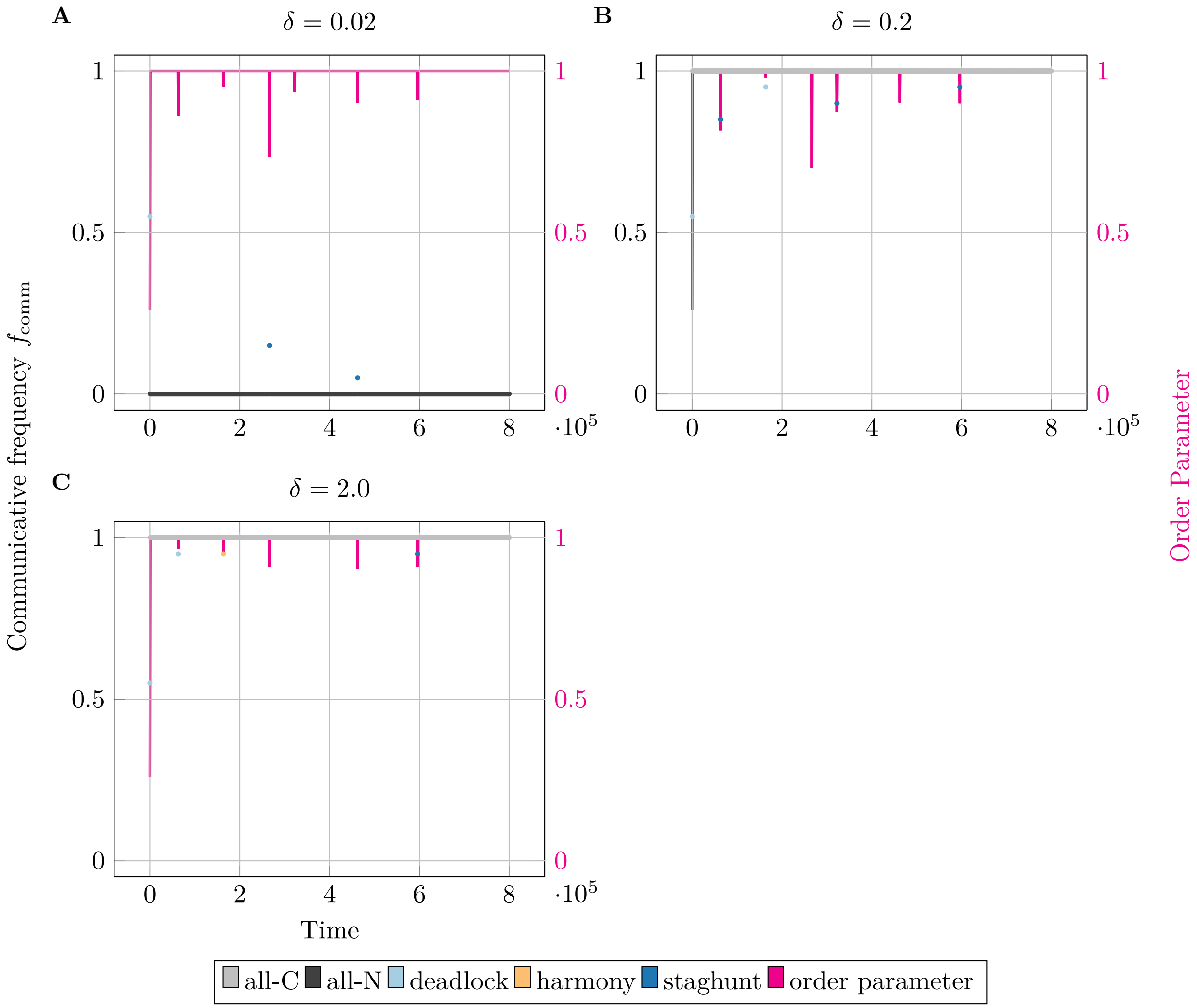

Fig B| Selection strength impact. Scatter plots of the instantaneous f_{comm} and order parameter ρ for different values of the selection strength δ, as shown in the subplot titles. The left vertical axes show the instantaneous f_{comm} as a function of time and are color-coded according to the plurality mixed game type as indicated in the legend. The right vertical axes give the order parameter ρ Scatter plots use the same axes and coloring as Fig. 3A-3C in the main text showing the communicative frequency, plurality mixed game-types, and order parameter. The symmetry breaking is $\alpha = 1$. The selection strengths shown are (A) $\delta = 0.02$, (B) $\delta = 0.2$, and (C) $\delta = 2.0$.

However, the sensitivity analysis shows that the system is very sensitive to the selection strength, with higher selection δ yielding stronger chimera-like states. Therefore, Fig. B displays time-series data from three values of δ: very weak selection 0.02, (baseline) weak selection 0.2, and strong selection 2.0. The other parameters are the usual values from the main text, and the symmetry breaking is $\alpha = 1.0$. Interestingly, the very-weak case $\delta = 0.02$ shows that the system is unable to maintain communication even in this strongly biased $\alpha = 1$ regime. Both the weak $\delta = 0.2$ and strong $\delta = 2.0$ cases successfully maintain communication. Furthermore, we see that the stronger selection $\delta = 2.0$ dampens some of the mutation-induced jumps, as is expected by stronger fitness-induced selection.

4 Phase *vs.* Strategy

We note that the communicative fraction is only sensitive to the players' strategies, so fixing the strategies would be uninformative, showing only the random initial distribution. Likewise, the chimera-like index is only sensitive to the players' phases, so fixing the phases would be uninformative, showing only the random initial distribution.

First, the (B) large chimera-like index of the fixed-strategy case shows that phase-evolution is a key part of chimera-like evolution. Likewise, the (D) similarity of the fixed-phase case—and hence, large deviation from the well-mixed case—demonstrates that strategy-evolution of *Caenorhabditis elegans* (*C. elegans*) alone introduces qualitatively new behavior compared to the well-mixed case. Finally, the baseline, co-evolving strategy- and phase-dynamic case differs from both the (B) fixed-strategy and (D) fixed-phase cases. This implies that the co-evolving phase and strategy used in the main text introduces novel dynamics not captured by either the phase or strategy alone.

References

1. Bruns B. Names for Games: Locating 2×2 Games. Games. 2015 Oct;6(4):495-520. doi:10.3390/g6040495.

2. Tripp EA, Fu F, Pauls SD. Evolutionary Kuramoto Dynamics. Proc R Soc B. 2022 Nov;289(1986):20220999. doi:10.1098/rspb.2022.0999.

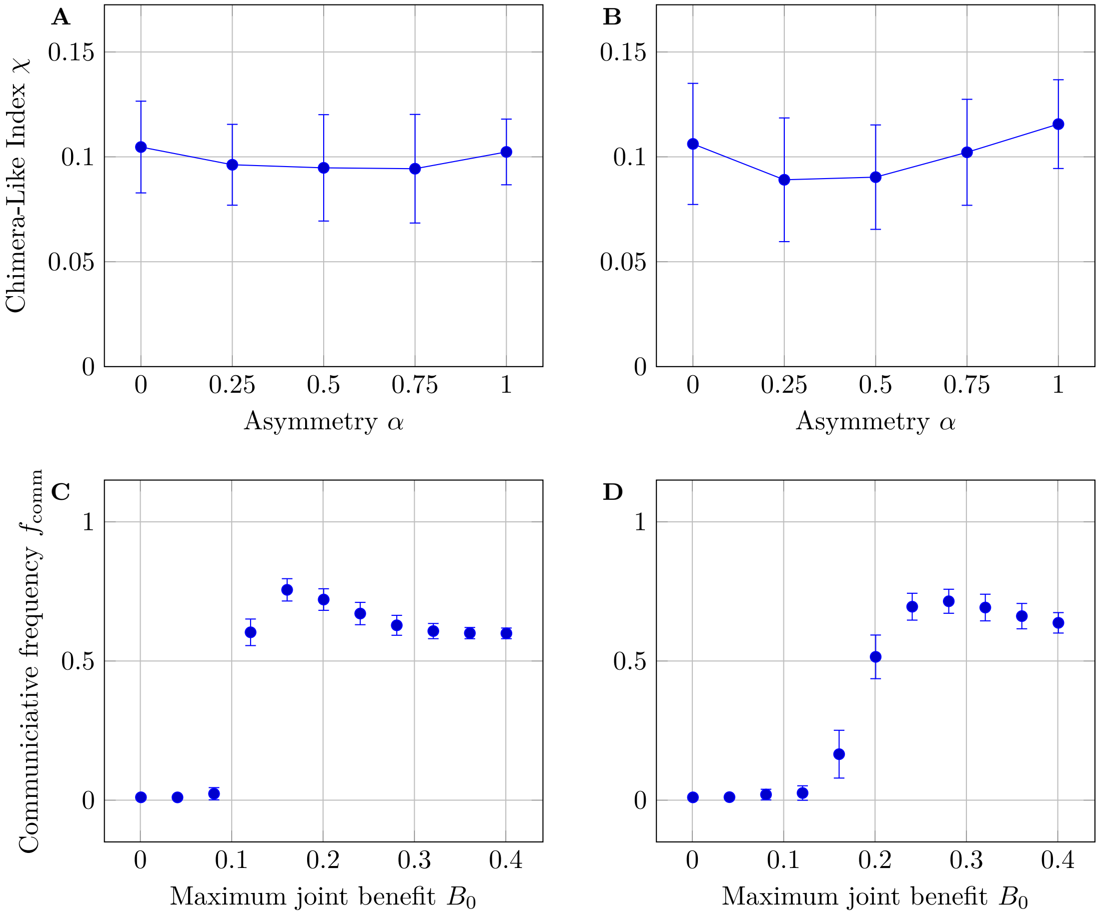

Fig C| Comparison of strategy vs phase evolution. Comparison of strategy vs phase evolution on the chimera-like index and communicative fraction. (A,B) The chimera-like index is shown as a function of asymmetry α. (A) is the baseline case with both strategy and phase updated according to the birth-death Moran process, while (B) keeps the C/N strategy of each player fixed to its random initial value but subjects its phase ϕ_i to the birth-death Moran process. The vertical error bars depict the standard deviation across ten seeds. (C,D) The communicative fraction f_{comm} as a function of the maximum joint benefit B_0. (C) is the baseline case with both strategy and phase updated according to the birth-death Moran process, while (D) has the phase ϕ_i of each player fixed to its random initial value while its C/N strategy is subject to the birth-death Moran process.
