# Supplementary material for: Evolutionary Kuramoto dynamics unravels origins of chimera states in neural populations
Source: PLoS Comput Biol. 2026 Apr 30;22(4):e1014214. doi: 10.1371/journal.pcbi.1014214 (PMC13152215; doi:10.1371/journal.pcbi.1014214)
Supplement: Attachment — Submitted filename: Response-Reviewer2.pdf [file pcbi.1014214.s006.pdf]

REF: #PCOMPBIOL-D-25-02369; REPLY TO: Reviewer 2

Evolutionary Kuramoto dynamics unravels origins of chimera states in neural populations

Thomas Zdyrski, Scott D. Pauls, and Feng Fu

We thank referee #2 for this thoughtful review of the manuscript which is reproduced below in dark grey Sans-serif font. A detailed reply to the referee's comments follow (in Serif font) and modifications to the manuscript are indented and set in *blue italics*–also included with the resubmission is a copy of the revised manuscript with these changes in blue.

1. The abstract: The abstract is pretty solid, but it jumps right into the model without much context on why chimera states matter in real brains maybe add a few sentences tying it back to cognition or disorders to hook readers more.

We have added additional motivation to the abstract on lines 10-15:

> *However, incomplete synchronization often produces chimera states , where coherent and incoherent dynamics coexist. Recent studies have suggested that these chimera states could be important in human cognitive organization. In particular, chimera states have been suggested as a regulator of cognitive integration and regulation with varying quality as humans age.*

2. I like how they extend the Kuramoto model with evolutionary game theory; it's a fresh take, especially adding asymmetry in payoffs. But is this asymmetry biologically justified? Like, do neurons really have "biased" communication like that in nature?

We thank the reviewer for the kind words. We have also further biological justification to the introduction on lines 107-114:

Furthermore, this payoff asymmetry between communicators (C) and non-communicators (N) models the inherent asymmetry of pre- and post-synaptic junctions: if the source neuron is N and the destination neuron is C, then the destination neuron's payoff is modulated by 2α, which represents its receptivity to other, non-local chemical neurotransmitters [26]; conversely, if the source neuron is C and the destination neuron is N, then the destination neuron's payoff is modulated by $2(1-\alpha)$, representing its ability to spontaneously fire in absence of upstream stimuli [27].

3. The parameter space section (Fig 2) is impressive, showing all these game types like snowdrift or deadlock. It enriches the discussion beyond just prisoner's dilemma, but the phase diagrams feel a tad crowded, perhaps simplify the legends or add a table summarizing key transitions.

We appreciate the reviewer's feedback. We have narrowed the legend to make it easier to read and added a table (**??**) calling out selected transitions to improve the clarity of **??**. Accompanying description was added to lines 155-159:

The table in Fig. 2C *shows some select transitions as $\Delta\phi_i$ varies from 0 to π. For instance, when $B_0/c = 1.5$—corresponding to the line of white dots in* Fig. 2A*—$\Delta\phi = 0$ starts in the chicken region and moves left, transitioning to dilemma and then staghunt. As $\Delta\phi$ increases from π to 2π, this process reverses.*

4. In the complete graphs results (Fig 3), the simulations match the analytic predictions nicely for well-mixed populations. The time-series plots show interesting fluctuations, especially with alpha=1 where it's all over the place. Wonder if stronger selection would stabilize it more?

We thank the reviewer for the kind words. We are having some difficulty understanding to which $\alpha = 1$ fluctuations the reviewer is referring in Fig. 3. If the reviewer is referring to the short, delta-like drops, we have clarified the influence of mutations on the plot in lines 235-242:

The order parameter and communicative frequency are shown for a single seed to demonstrate the mutation-induced jumps for a single simulation. The mutation rate $\mu = 1 \times 10^{-4}$ means that a mutation occurs, on average, every 1×10^4 *time steps; for*

> *the* 8×10^5 *time steps, we therefore expect* 80 *mutations. These mutations cause the observed dips in the order parameter. But we note that there are significantly fewer than* 80 *dips shown. The quick extinction of mutant invaders means that they are often missed when we* 80×*-decimate the time-series for plotting, only observing* ≈ 6 *visible mutations.*

Additionally, the reviewer mentions wondering about the effect of stronger selection. We have added a new figure to the supplemental information showing the timeseries data for weaker and stronger values of the selection strength δ. We apologize if we have misinterpreted the reviewer's comment, and we are more than happy to further augment the analysis if further clarifications are needed.

5. Applying this to C. elegans connectome is the highlight for me, using a real neural network adds credibility. But they mention 302 neurons; did they account for different neuron types (sensory, motor) or just treat them all the same? That could be a limitation.

The 302 neurons includes sensory neurons, interneurons, and motor neurons. For simplicity, we have treated all of these neuron types identically. We have updated the text (lines 261-264) to clarify this:

> *For reference, Fig. 4D shows the* $N = 300$ *nodes and the directed edges (we exclude the two unconnected neurons CANL/CANR). We treat all* Caenorhabditis elegans *(*C. elegans*) neurons identically and do not differentiate between sensory neurons, interneurons, and motor neurons.*

Furthermore, we have highlighted this potential limitation and discussed potential future improvements on lines 458-461:

> *One potential limitation of this work is the identical treatment of different neuron types. Potential extensions could modulate the* B_0/c*,* β_0/c*, and* α *parameters of the different neuron types separately to better account for the specialization of these cells.*

6. The model assumes phases are discrete (m=20), which is fine for computation, but in real neurons, phases are continuous. How sensitive are the chimera states to m? A quick sensitivity analysis would help.

We have added a convergence analysis to Section 4.9 with a new figure Fig. 10 validating that $m = 20$ is an good approximation of the continuous phase by comparing to analyzing the communicative fraction, as described in lines 199-202:

> *The $m = 20$ discrete phases approximate a continuous phase distribution; Fig. 10 depicts a convergence analysis showing that the communicative fraction has converged already for $m = 20$, verifying that this is an acceptable approximation.*

7. Payoff structure with alpha biasing towards communicators makes sense intuitively, but the values (B0=0.15, c=0.1) seem arbitrary. Where do these come from? Any link to experimental neural costs/benefits?

We added a new section (Section 2.3) discussing the driving parameter ratios in our model in lines 178-186. Additionally, we clarified why these particular parameters were chosen on lines 187-199:

> *The ratio $B_0/c \approx 2.1$ is the critical joint-benefit benefit strength at which the communicative fraction equals $1/2$ when in the absence of asymmetry(*i.e., *$\alpha = 1/2$) [5]. Therefore, we follow [5] and choose $B_0/c = 1.5$ slightly less than the critical point to slightly bias the system towards non-communicativeness. Likewise, we choose $\beta_0/B_0 = 0.95$ since we biologically expect mixed NC communication to be less rewarding than joint CC communication. Both the mutation rate μ and selection strength δ are chosen to be small to allow for analytic approximations (see the supplementary materials). While we fix a common set of parameters in our results for simplicity, we also performed a sensitivity analysis in Section 4.9 to analyze the influence of varying these parameters. The main results (the chimera-like index χ) are largely insensitive to changes in most of these parameters. The only sensitive parameter is the selection strength δ, with stronger δ increasing the chimera-like effect. However, we retain the smaller δ for analytic tractability.*

Finally, we added a new section, Section 4.9, containing a sensitivity analysis of the chimera-like index against the relevant parameters. This includes a new figure Fig. 9C showing how the different parameters affect the chimera-like index.

8. The author summary is concise and accessible, good for non-experts. It emphasizes the bridge from small-scale neurons to large-scale dynamics, which is spot on for computational biology.

We thank the reviewer for the kind words.

9. Data availability is great.they've got Zenodo links and GitHub for code, even an interactive webpage. That's transparent and reproducible, kudos. But check if the code runs smoothly; I didn't test it, but it looks well-organized.

We appreciate the reviewers compliment. We have also updated the GitHub and Zenodo code bases with the changes recommended by the reviewers.

10. Overall, the paper's novel in combining EGT with Kuramoto on directed graphs, and it sheds light on chimeras. Minor revisions for clarity on assumptions and maybe more bio validation, but I'd recommend acceptance and it's a strong contribution.

We sincerely appreciate the reviewer's advice and feedback. The reviewers suggestions were exceedingly helpful in strengthening the paper. We appreciate the reviewer's time and efforts in helping us to strengthen our manuscript.
